# Dispersion braiding and band knots in plasmonic arrays with broken symmetries

**DOI:** 10.1515/nanoph-2023-0062

**Published:** 2023-03-30

**Authors:** Shixiong Yin, Andrea Alù

**Affiliations:** Department of Electrical Engineering, City College of The City University of New York, New York 10031, USA; Photonics Initiative, Advanced Science Research Center, The City University of New York, New York 10031, USA; Physics Program, Graduate Center, City University of New York, New York 10016, USA

**Keywords:** nanophotonics, nonlocal photonics, plasma, plasmonic

## Abstract

Periodic arrays can support highly nontrivial modal dispersion, stemming from the interplay between localized resonances of the array elements and distributed resonances supported by the lattice. Recently, intentional defects in the periodicity, i.e., broken *in situ* symmetries, have been attracting significant attention as a powerful degree of freedom for dispersion control. Here we explore highly nontrivial dispersion features in the resonant response of linear arrays of plasmonic particles, including the emergence of braiding and band knots caused by band folding. We show that these phenomena can be achieved within simple dipolar arrays for which we can derive closed-form expressions for the dispersion relation. These phenomena showcase powerful opportunities stemming from broken symmetries for extreme dispersion engineering, with a wide range of applications, from plasma physics to topological wave phenomena. Our theoretical model can also be generalized to higher dimensions to explore higher-order symmetries, e.g., glide symmetry and quasi-periodicity.

## 1 Introduction

In wave physics, periodic artificial media and metamaterials have become a powerful platform to manipulate wave-matter interactions. Among them, plasmonic arrays have been widely studied for enhancing optical functionalities, including sensing [1–3], Raman scattering [4–6], fluorescence [7, 8], valley polarization control [9–11], etc. In parallel, integrating plasma into metamaterials has led to more exotic and tunable electromagnetic devices, e.g., circulators [12], directional scatterers [13] and waveguides, as well as demultiplexers [14]. Recently, tailored perturbations within the lattice of otherwise periodic media have revealed extreme wave-matter interactions, such as bound states in the continuum [15–17], parity-time symmetries [18], and exceptional points [19], which pave the path to exploring nonlocal photonics and flat optics [20–22]. As a remarkable example, if a regular perturbation is introduced in a periodic array, for which the ratio of the modulation period over the lattice of the unmodulated array is a rational number *p*/*q* with *p* and *q* being positive integers, the dispersion curves fold and split into *q* sub-bands [23]. These splitting non-orthogonal bands feature exotic dispersions features, with rich topological implications, e.g., Anderson localization [24], braiding [25], and windings [26] of the non-Hermitian band structures.

Powerful broken spatial symmetries in one-dimensional (1D) structures include glide symmetry and screw symmetry [27]. Wave propagation in systems with such asymmetries can be studied as eigenvalue problems for corresponding symmetry operators, revealing exciting physics and various applications reported in particular by the microwave community [28–30], including broadband lenses [31], microwave edge modes [32], and high refractive index electromagnetic devices [33]. In topological physics, the Su–Schrieffer–Heeger (SSH) model, first proposed to model polyacetylene molecules [34], can also be treated as a 1D array with *in situ* asymmetries, for which the energy levels split into two braids [35, 36].

Here, we analyze packed plasmonic or plasma particle arrays with broken *in situ* symmetry. Inspired by the SSH model, we first consider an infinite 1D array of polarizable particles in which every other particle is slightly perturbed. Then, using coupled dipole approximations [37–39], we derive closed-form dispersion relations and validate them with numerical examples for plasma spheres. As expected, we observe band folding as the broken symmetries are introduced. An interesting regime emerges when the particles are closely packed and support highly nonlocal responses. In this scenario, the band folding introduces a highly exotic form of self-anticrossing of the dispersion band, inducing a knot (unlink) in reciprocal space. In this scenario, a dark plasmonic mode is strongly coupled with a much brighter modal field distribution near the knot. In addition, the highly confined surface plasmon modes with large momenta in the unperturbed array evolve into highly directive leaky modes under broken symmetries, whose degree of leakage can be controlled through the perturbation strength. We also generalize these results to polymerization, where every *N* > 2 individual perturbations form a new meta-atom. In this scenario, the arbitrary *in situ* asymmetry is modeled as an *N*th order eigenvalue problem, which offers again closed-form expressions for the dispersion equation. The theoretical model introduced here offers powerful tools to study the effect of broken symmetries in dispersive arrays, leveraging the interplay of localized resonances, nonlocal lattice phenomena and broken symmetries. Extensions to higher dimensions, e.g., glide/screw symmetry and quasi-periodic media may open further opportunities, with implications also for the emerging field of topological photonics [40].

## 2 Dispersion relations for dimerized plasmonic arrays

A 1D periodic array of polarizable particles can be characterized as a chain of dipole moments under the assumption that each unit element is much smaller than the wavelength in the background medium and hence dominated by a dipolar scattering response. The array supports collective resonant eigenmodes whose dispersion relation under the Bloch boundary condition can be written in a compact form as [37–39]

(1) $$\det\left(\alpha_{\text{ee}}^{-1}I-\overset{↔}{G}\cdot {\hat{x}}_{i}\cdot {\hat{x}}_{j}\right)=0,$$

where *α*_ee_ is the electric polarizability of the individual particle (assumed isotropic here for sake of simplicity; extensions to anisotropic responses are straightforward). **I** is the identity matrix and $\overset{↔}{G}$ is the square matrix of dyadic Green’s functions. Due to symmetry, we can decompose the eigenmode solutions into longitudinal (with dipole moments along the array) and transverse (orthogonal to the array) polarizations, for which the unit vectors ${\hat{x}}_{i}$ and ${\hat{x}}_{j}$ are directed either along longitudinal or transverse directions. Equation (1) captures many well-established phenomena, from plasmonic lattice resonances [41] to Woods anomalies [42], and in principle can describe complex lattice resonances, e.g., involving periodic perturbations, under the dipole approximation [43–45]. Here, we start by considering a fully periodic array with periodicity *d* lying along the *x*-axis and with dipole polarization ${\hat{x}}_{p}$ , then ${\hat{x}}_{p}\cdot \hat{x}=1$ for longitudinally polarized dipoles and ${\hat{x}}_{p}\cdot \hat{x}=0$ for the transverse case. Equation (1) under the time-harmonic convention e^−i*ωt*^ simplifies to [39]

(2) $$\begin{array}{ll} {\bar{\alpha_{\text{ee}}}}^{-1} & =\frac{3}{2}{\bar{d}}^{-3}\left(3{\hat{x}}_{p}\cdot \hat{x}-1\right)\left[f_{3}\left(\bar{\beta },\bar{d}\right)-\text{i}\bar{d}f_{2}\left(\bar{\beta },\bar{d}\right)\right.\\ & \;\left.-\left(1-{\hat{x}}_{p}\cdot \hat{x}\right){\bar{d}}^{2}f_{1}\left(\bar{\beta },\bar{d}\right)\right], \end{array}$$

where $f_{\ell }\left(\bar{\beta },\bar{d}\right)=Li_{\ell }\left(e^{\text{i}\left(1+\bar{\beta }\right)\bar{d}}\right)+Li_{\ell }\left(e^{\text{i}\left(1-\bar{\beta }\right)\bar{d}}\right)$ and $Li_{\ell }\left(\cdot \right)$ is the polylogarithm of *ℓ*th order. Note that all involved quantities are normalized here: $\bar{d}=kd$ , $\bar{\beta }=\beta /k$ , and $\bar{\alpha_{\text{ee}}}=\alpha_{\text{ee}}k^{3}/\left(6\pi \varepsilon_{0}\right)$ , where *k* = *ω*/*c*, *c*, and *ɛ*_0_ are the wavenumber, speed of light, and permittivity in the background, respectively, and *β* is the momentum along the longitudinal direction for the eigenmode.

In the following, we assume *β* to be a real quantity while *ω* (therefore *k*) is in general complex, in the presence of material loss and/or possible radiation leakage. $f_{1}\left(\bar{\beta },\bar{d}\right)$ inside the bracket in Eq. (2) is associated with the far-field interactions among the dipoles, derived from the summation over ∼1/r terms [46].

Interestingly, this contribution only occurs for transversely-polarized resonant particles as $1-{\hat{x}}_{p}\cdot \hat{x}\neq 0$ , implying strong nonlocal effects. For sufficiently tight (*kd* < 1.517 in our scenario, see Supplementary Materials) arrays, the band diagram can support a stationary point in the first Brillouin zone, due to such strong nonlocal effects coupled to the plasmonic resonance of the inclusions [39]. In fact, the group velocity becomes zero at this point and then flips to a negative value within the first Brillouin zone. It should be stressed again that this is a highly nonlocal phenomenon, since the bending of the dispersion implies the occurrence of two distinct eigenmodes with the same transverse polarization at the same frequency. These features have been explored to realize negative-index metamaterials [47]. In the following, we explore the introduction of long-range perturbations in this regime, introducing band folding in this highly nonlocal regime of wave propagation.

**Figure 1:**
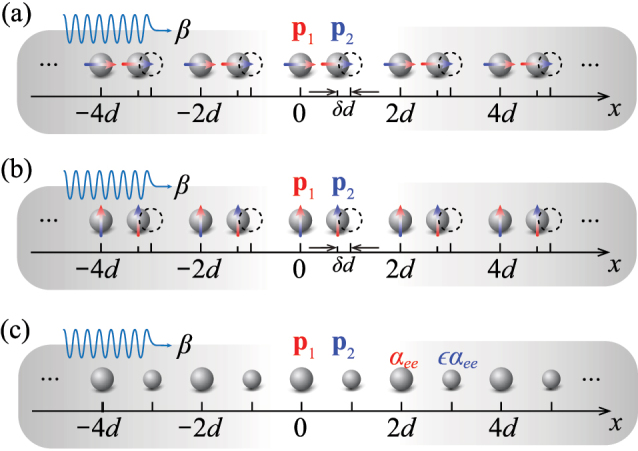
1D arrays of polarizable particles with period *d* and broken *in situ* symmetries. *β* is the longitudinal wave number. A longitudinal translation *δd* is imposed on every other particle in (a) longitudinally polarized particles, and (b) transversely polarized particles. Black-dashed outlines illustrate the initial positions of unperturbed particles. **p**_1_ and **p**_2_ are two individual dipole moments in the new unit cell of period 2*d*. Panel (c) describes the broken symmetries by alternating the electric polarizability of every other particles by a scale factor *ε*.

As a first perturbation scheme, we break *in situ* symmetries of the plasmonic array by introducing a translation on every other particle by a relative distance *δ* normalized to the original period *d*, as sketched in Figure 1(a) and (b). The perturbed plasmonic array is still a periodic structure with period 2*d*. Thus, the first Brillouin zone is now bounded by *βd* = *π*/2. The intra- and inter-coupling between the dipoles can be modeled as two distinct dipole moments **p**_1_ and **p**_2_, as shown in Figure 1 by differently-colored arrows. As another form of perturbation, we also consider imposing a perturbation on the electric polarizability, without altering the position. For instance, as shown in Figure 1(c), every other particle in the plasmonic array is shrunk, leading to two distinct polarizabilities *α*_ee_ and *ε* *α*_ee_. Mathematically, the dyadic Green’s function matrix $\overset{↔}{G}$ in Eq. (1) then becomes of rank 2, and its off-diagonal terms are associated with the perturbation *δ*. In this scenario, each unit cell includes two individual dipole moments, which are related to the local fields through the electric polarizability:

(3) $$p=\left(\begin{array}{cc} 1\; & 0\\ 0\; & \epsilon \end{array}\right)\alpha_{\text{ee}}E_{\text{loc}}$$

with $p={\left(p_{1},p_{2}\right)}^{\text{T}}$ and $E_{\text{loc}}={\left(E_{\text{loc},1},E_{\text{loc},2}\right)}^{\text{T}}$ where the superscript T denotes the transpose. Evidently, *ε* = 1 for the scenarios shown in Figure 1(a) and (b) where all the particles are identical. The local fields can be related through the Green’s functions to the dipole moments, as discussed in the Supplementary Material. We obtain

(4) $$\left(\begin{array}{cc} 1 & 0\;\\ 0\; & \epsilon \end{array}\right)\left(\begin{array}{cc} {\bar{G}}_{11}\; & {\bar{G}}_{12}\;\\ G_{21} & {\bar{G}}_{22} \end{array}\right)\left(\begin{array}{c} p_{1}\\ p_{2} \end{array}\right)={\bar{\alpha_{\text{ee}}}}^{-1}\left(\begin{array}{c} p_{1}\\ p_{2} \end{array}\right),$$

and the dispersion relation is then given explicitly by

(5) (5) $${\bar{\alpha_{\text{ee}}}}^{-1}\left(\omega \right)=\frac{1+\epsilon }{2}{\bar{G}}_{11}\pm \sqrt{{\bar{G}}_{12}{\bar{G}}_{21}+{\left(\frac{1-\epsilon }{2}{\bar{G}}_{11}\right)}^{2}}.$$

${\bar{G}}_{ij}\left(\omega ,\beta \right)$ with *i*, *j* = 1, 2 are the normalized Green’s functions associated with Lerch transcendental $\Phi \left(z,\ell ,x\right)=\sum_{n=0}^{\infty }z^{n}{\left(n+x\right)}^{-\ell }$ [48], which are derived in the Supplementary Materials.

We generally find two complex eigenfrequencies by choosing two branches of (“±”) of the square-root function in Eq. (5), suggesting the emergence of two braids of the dispersion curves in the halved Brillouin zone 0 ∼ *π*/(2*d*). Meanwhile, passivity enforces that $Im\left\{{\bar{\alpha_{\text{ee}}}}^{-1}\right\}=-1$ for any lossless dipolar particles, corresponding to the radiation from an individual scatterer [49]. Hence, in the region under the light line [ $Re\left\{\omega \right\}<c\beta$ ] where *ω* is real and there is no net radiation loss from the lattice to free space, $Im\left\{{\bar{\alpha_{\text{ee}}}}^{-1}\right\}=-1$ should still hold [39, 46]. For the eigenfrequencies above the light line [ $Re\left\{\omega \right\}>c\beta$ ], however, an additional term is expected to add on to $Im\left\{{\bar{\alpha_{\text{ee}}}}^{-1}\right\}$ evaluated at complex *ω*, indicating the interference of individual dipole radiation that produces a leaky mode [39].

## 3 Dispersion braiding and band knots

In this section, we numerically demonstrate the exotic dispersion features emerging when band braiding is combined with strong nonlocality. In the following examples, we choose lossless plasmonic nanospheres to constitute the plasmonic arrays. The dynamic polarizability of such a lossless plasmonic sphere then follows [50]

(6) $$\alpha_{\text{ee}}^{-1}=\frac{1}{4\pi \varepsilon_{0}a^{3}}\frac{\varepsilon \left(\omega \right)+2\varepsilon_{0}}{\varepsilon \left(\omega \right)-\varepsilon_{0}}-\text{i}\frac{k^{3}}{6\pi \varepsilon_{0}}.$$

*ɛ* is the permittivity of the sphere, and is assumed to follow a Drude dispersion $\varepsilon \left(\omega \right)=\varepsilon_{0}\left(1-3\omega_{0}^{2}/\omega^{2}\right)$ , such that an isolated sphere experiences the dipole resonance with $\varepsilon \left(\omega_{0}\right)=-2\varepsilon_{0}$ . The radius of the sphere is set to deep subwavelength as *a* = 0.05 × 2*πc*/*ω*_0_, and the center-to-center distance between two spheres in the unperturbed periodic array is *d* = 3*a*. Rigorous models of the polarizability for more complicated geometries may also be employed, e.g., the one for core–shell structures derived from Mie theory as discussed in Ref. [51].

**Figure 2:**
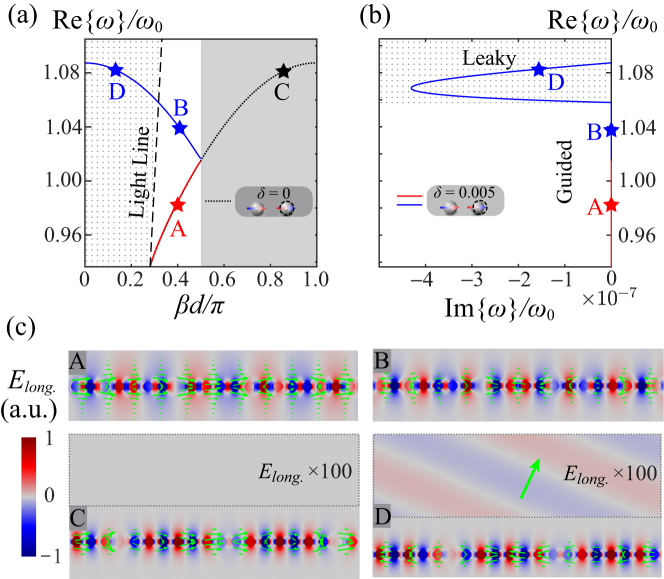
Dispersion braiding for longitudinally polarized plasmonic arrays. (a) Dispersion diagrams of both unperturbed (black dotted curve) and the dimerized (red and blue ones, resulting from translating every other sphere by the relative distance *δ* = 0.005) lattices in the longitudinal polarization. The dot-shaded region highlights the leaky-mode region (black-dashed line). (b) Real part of eigenfrequencies versus their imaginary part. (c) Simulated field distributions of selected eigenstates as labeled in panel (a). Green arrows show the time-averaged Poynting vectors.

In this assumed configuration, the unperturbed plasmonic array in the longitudinal polarization supports a forward-guided mode (black curve), as shown in Figure 2(a). Here we break the original periodicity by translating every other sphere by a relative distance *δ* = 0.005, determining the split and fold effect at the center of the original Brillouin zone. The lower braid (red curve) is almost unaffected by the perturbation, except for the splitting around *β* = *π*/(2*d*), whereas the upper braid (blue curve) crosses over the light line (black dashed line) and is converted into a leaky-wave branch. Despite the lossless nature of the array, the imaginary part of *ω*, as shown in Figure 2(b), becomes nonzero inside the dotted region, corresponding to that above the light cone in Figure 2(a) [ $Re\left\{\omega \right\}>c\beta$ ], due to the emergence of radiation loss supported by the broken symmetry. Figure 2(c) shows the simulated longitudinal component of the electric field for the longitudinally-polarized eigenmodes, embedded with time-averaged Poynting vectors (green arrows) as retrieved from COMSOL Multiphysics, corresponding to the selected states labeled in Figure 2(a). Eigenstate B, compared to A, shares the same longitudinal momentum and thus the same spatial periodicity in their field distributions, but features backward power flows as expected by a negative group velocity in Figure 2(a). With a larger *β* the unperturbed lattice supports a more confined guided mode C traveling forward, where the field decays rapidly away from the array. Similarly, the small perturbation converts C to a backward mode D, with almost identical near-field distributions. However, as expected from non-zero Im{*ω*} in Figure 2(b), the presence of the perturbation would now leak out a small portion of the original forward-guided mode in the form of radiation. As shown in the dashed box that visually magnifies the field, this leaky mode radiates power to the far field in the opposite direction to its near-field power flow, forming a proper backward leaky mode [52, 53].

**Figure 3:**
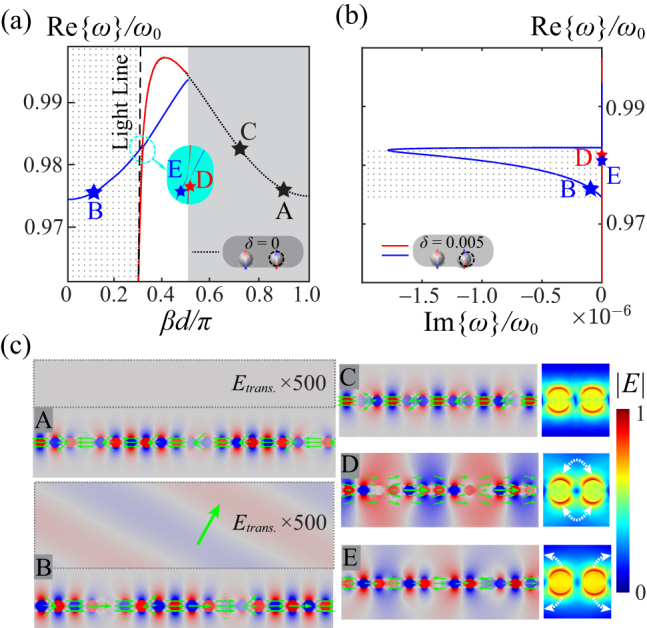
Similar as in Figure 2, but for transverse polarization. (a) Dispersion diagrams before (black dotted curve, partially covered by the red one for *βd* < 0.5*π*) and after (red and blue ones) braiding, due to a relative translation distance *δ* = 0.005. (b) Real part of eigenfrequencies versus their imaginary part. (c) Modal field distributions of selected eigenstates as labeled in panel (a). The insets in the plots of C–E zoom into the dimer colored with their corresponding field amplitudes.

The situation becomes nontrivial for transversely polarized arrays, as shown in Figure 3: the high nonlocality in the unperturbed scenario bends the band diagram, arousing a guided backward mode in the first Brillouin zone, as depicted by the black dashed curve in Figure 3(a). For instance, state A on this curve possesses a large momentum and a negative group velocity, whose modal field is tightly confined to the lattice. Once the perturbation is introduced, band braiding is also observed, as shown in the Brillouin zone for the perturbed array (0 < *βd* < 0.5*π*) in Figure 3(b). The red braid follows nearly the same dispersion as the unperturbed one until the splitting at *βd* = 0.5*π*. The blue braid then emerges by folding the unperturbed dispersion curve (black dotted curve). Similar to the longitudinal scenario discussed above, the guided mode A now evolves to a leaky mode B with opposite negative group velocity, as well as non-zero Im{*ω*} shown in Figure 3(b), forming an improper forward leaky wave [53] as magnified in the dashed box in Figure 3(c)—state B. In Figure 3(b), note that for 0.974 < *ω*/*ω*_0_ < 0.983 (dot-dashed region) both leaky (e.g., state B) and guided modes (e.g., states D and E) are supported by the perturbed lattice. The discontinuity of Im{*ω*} around *ω* = 0.983*ω*_0_ is due to the fact that eigenstate is mathematically ill-defined on the light line, stemming an essential singularity of the first-order Lerch transcendental coinciding with the branch cut *β* = *ω*/*c* of the Green’s function. More details about the singularity are included in the Supplementary Materials.

More interestingly, the presence of the stationary point in the reciprocal space due to the strong nonlocal effects enforces the blue braid to anti-cross with the red one, as shown in the inset in Figure 3(a). This anti-crossing forms a trivial topological knot—unlink—in the 3D space of complex energy (*ω*) versus the Bloch wavenumber *β*, as classified in the case of non-Hermitian band structures [54] (see Supplemental Materials for an example with plasmonic arrays considering material loss, where all the eigenfrequencies become complex and thus span non-Hermitian band structures). Since the anti-crossing knot is quite close to the light line, the modal field hybridizes the forward quasi-plane wave and the tightly guided mode of the unperturbed case. The eigenstates D and E near the anti-crossing are shown in Figure 3(c): state D on the red braid is relatively bright, consistent with the quasi-plane wave close to the light line of the unperturbed array, whereas state E on the blue braid acquires the features of the unperturbed counterpart state C, much more tightly bound to the array. As shown by the white arrows in the adjacent subplots, state D exhibits stronger intra-cell coupling, whereas the dimer in state E features slightly more inter-cell interactions with its neighbors. Overall, significant similarities in the modal field amplitudes |*E*| can be observed among states C, D, and E. We reiterate that the braiding and associated band knots reported here are highly nonlocal phenomena, as a result of the interplay among the individual plasmonic resonance, periodic perturbations and collective response the entire array. Hence, small aperiodic random disorder and imperfections, which can be effectively modeled as additional “loss” in the system [55], do not hinder the emergence of the knots in reciprocal space. See Supplementary Materials for discussions about the effect of material loss on these phenomena.

**Figure 4:**
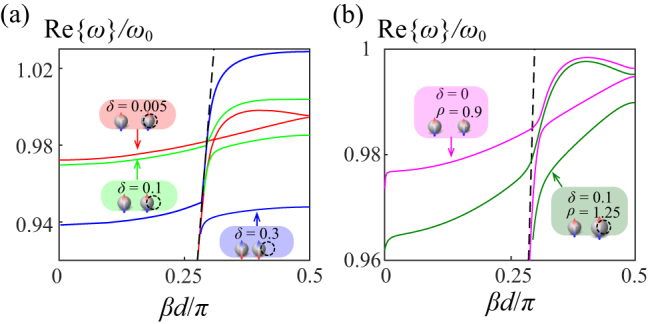
Dispersion diagrams for the plasmonic arrays with different types of broken symmetries. (a) Dispersion diagrams for different translational perturbation in position. (b) Dispersion diagrams for the cases with broken symmetries in polarizability by shrinking every other sphere (pink), and in both position and polarizability by expanding every other sphere (green), respectively.

As we increase the translation perturbation in position, the anti-crossing knot is expected to loosen up, as shown in Figure 4(a). The forward leaky modes emerge as well in the scenarios with larger perturbation *δ* = 0.1 (green curve) and 0.3 (blue curve), even though the stationary point below the light line vanishes. Another type of *in situ* asymmetry—perturbing the polarizabilities—is demonstrated here by altering the radius of every other sphere in the transversely-polarized lattice by a scaling factor *ρ*. For instance, the inset with a pink background in Figure 4(b) illustrates an example of *ρ* = 0.9 where the radius of the sphere on the right is shrunk to 90% of the one of the spheres on the left. The dispersion in this case is depicted by the pink curve. More generally, we can even perturb the array in both *in situ* positions and polarizabilities of the spheres, as demonstrated in the green inset in Figure 4(b), with every other sphere expanded by 25% on its original radius (*ρ* = 1.25) and translated by 10% of the original period (*δ* = 0.1) at the same time. The bending features and the stationary points under the light line survive in both cases, while the leaky mode above the light line has larger positive group velocities compared to the one with translational *in situ* asymmetry in position only.

## 4 Polymerization

**Figure 5:**
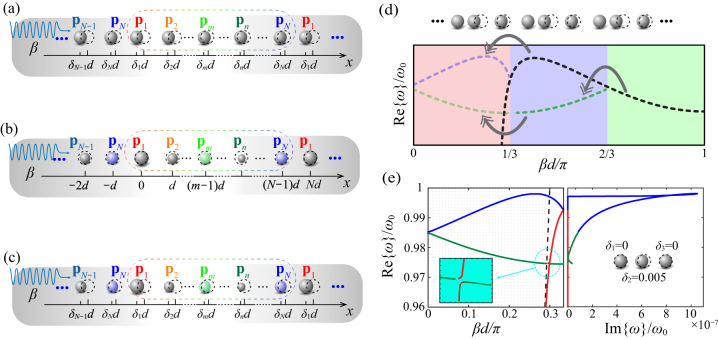
Polymerized lattices with broken symmetries: (a) In position, (b) in polarizability, and (c) in both position and polarizability. The round dashed box illustrates the long-range unit cell, including *N* particles with dipole moments from **p**_1_ through **p**_*N*_. The perturbations on polarizabilities are represented by alternating radii and changing materials (colors). (d) Illustration of band folding and braiding for a trimerized lattice with each unite cell enclosing three different dipole moments **p**_1,2,3_. (e) A numerical example for the dispersion of a trimerized array by translating only the middle particle with *δ*_2_ = 0.005.

Our theoretical model so far has considered dimerized forms of perturbation, yet it can be readily generalized to polymerization. As sketched in Figure 5(a)–(c), we consider a long-range-ordered array where every *N* particles constitute a large period, with either translation in their positions [panel (a)] or alternations on polarizabilities [panel (b)], or both at the same time [panel (c)]. In this case, we define the symmetry-breaking vectors as $\Delta ={\left[-\delta_{1},1-\delta_{2},\ldots ,N-1-\delta_{N}\right]}^{\text{T}}$ and $\chi ={\left[\varepsilon_{1},\varepsilon_{2},\ldots ,\varepsilon_{N}\right]}^{\text{T}}$ , such that the positions and polarizabilities of *N* particles in one period (every *N* particles) are

(7) $$x=x_{0}-\Delta d=d{\left[-\delta_{1},1-\delta_{2},\ldots ,N-1-\delta_{N}\right]}^{\text{T}},$$

(8) (8) $${\left[\alpha_{\text{ee},1},\alpha_{\text{ee},2},\ldots ,\alpha_{\text{ee},N}\right]}^{\text{T}}=\alpha_{\text{ee}}\chi =\alpha_{\text{ee}}{\left[\epsilon_{1},\epsilon_{2},\ldots ,\epsilon_{N}\right]}^{\text{T}}.$$

where **x** denotes the relative locations inside one period to those of the unperturbed array **x**_0_. Note that both **Δ** and ***χ*** feature cyclic and translation symmetry, i.e., one may assign an arbitrary particle with index “1” and could also add an arbitrary constant between 0 and 1 to **Δ**. Finally, we generalize the dispersion equation for arbitrarily broken symmetry in the compact form

(9) $$\det\left[{\bar{\alpha_{\text{ee}}}}^{-1}I-\mathrm{diag}\left(\chi \right)\bar{G}\right]=0,$$

where **I** is an *N*-by-*N* identity matrix and diag(***χ***) is a diagonal matrix with diagonal entries correspond to the elements of ***χ***. The expression of each entry ${\bar{G}}_{mn}$ is derived in the Supplementary Materials, as a function of the vector Δ, relating the dipole moment of the *m*th particle to the contributions from all the dipoles with index *m*.

Analogous to the band folding once in the dimerized scenarios, periodically perturbing every *N* particles braids the band structure of the unperturbed lattice *N* − 1 times, shrinking the first Brillouin zone to *βd* < *π*/*N*. For instance, consider a lattice with periodically broken symmetries whose unit cell encloses three distinct dipole moments **p**_1,2,3_, as shown in Figure 5(d). We expect the original unperturbed band (black dashed curve) to be folded twice: one with respect to *βd*/*π* = 2/3 and another to *βd*/*π* = 1/3, illustrated by the gray arrows. Intuitively, the final band structure containing three braids falls into the red-shaded region where 0 < *βd*/*π* < 1/3. To demonstrate this numerically, we choose to perturb every other two spherical particles with the same size and Drude dispersion as above, by translating a relative distance *δ*_2_ = 0.005. The braided dispersion curves are shown in Figure 5(e), where both backward and forward leaky modes appear above the light line. The stationary point now lives above the light line on the blue braid, indicating a leaky wave supported by the lattice resonance with zero group velocity. An anti-crossing is also observed in this case, yet now between the forward-guided mode (red braid) and a backward mode (green braid). Notice that in the subpanel on the right of Figure 5(e), discontinuities in Im{*ω*} occur whenever the bands cross the light line, originating from the singularity in the Green’s functions as discussed above.

## 5 Conclusions

In this paper, we explored the dispersion of arrays of plasma or plasmonic particles with periodically broken symmetries using an analytical model that supports closed-form dispersion relations. Anti-crossing knots are observed in reciprocal space for transversely polarized arrays when broken symmetries affect strongly nonlocal phenomena in the array. Numerical simulations validate our theoretical predictions and demonstrate exciting opportunities for dispersion engineering, both for guided and radiation modes, stemming from nonlocal phenomena coupled to broken symmetries. We have also generalized our theoretical results to broken symmetries of any kind and any order, making it possible to analyze arbitrary spatial asymmetries of any long-range order. Our theoretical model can be extended to model more advanced symmetry classes supporting exotic topological phenomena, e.g., higher-order topological insulators protected by glide symmetry [56], as well as Hofstadter butterfly spectra in parameter space supported by quasi-periodic structures [57].

## Supplementary Material

Supplementary Material Details
