# Supplementary material for: Dispersion braiding and band knots in plasmonic arrays with broken symmetries
Source: Nanophotonics. 2023 Mar 30;12(14):2963–71. doi: 10.1515/nanoph-2023-0062 (PMC11614336; doi:10.1515/nanoph-2023-0062)
Supplement: Supplementary file 1 — Supplementary Material Details [file j_nanoph-2023-0062_suppl_001.pdf]

Supplementary Materials for "Dispersion Braiding and Band Knot in Plasmonic Arrays with Broken symmetries"

Shixiong Yin[1,2] and Andrea Alù*[1,2,3]

[1]Department of Electrical Engineering, City College of The City University of New York, New York 10031, USA

[2]Photonics Initiative, Advanced Science Research Center, The City University of New York, New York 10031, USA

[3]Physics Program, Graduate Center, City University of New York, New York 10016, USA

1. Group velocity and stationary point in the reciprocal space

The real part of the inverse polarizability $\mathrm{Re}\{\alpha_{ee}^{-1}\}$ is frequency (ω) dependent. Hence, it is a composite function of β in the reciprocal space. The chain rule of differentiation leads to $\partial[\mathrm{Re}\{\alpha_{ee}^{-1}\}]/\partial\beta = (\partial[\mathrm{Re}\{\alpha_{ee}^{-1}\}]/\partial\omega)(\partial\omega/\partial\beta)$, which derives the group velocity:

$$v_g = \frac{\partial\omega}{\partial\beta} = \frac{\partial Re\{\alpha_{ee}^{-1}\}}{\partial\beta} / \frac{\partial Re\{\alpha_{ee}^{-1}\}}{\partial\omega}. \tag{S1}$$

It equals zero if and only if the numerator of Eq. (S1) is zero. For transversely-polarized 1D periodic plasmonic array, the stationary point (zero group velocity) occurs when $\partial\mathrm{Re}\{\overline{\alpha_{ee}}^{-1}\}/\partial\bar{\beta} = 0$ [1], where $\bar{\beta}$ and $\overline{\alpha_{ee}}^{-1}$ are the normalized quantities as defined in the main text. By taking the derivative over $\bar{\beta}$ on Eq. (2) in the main text and equating it to zero, we find

$$\begin{aligned}&\mathrm{Cl}_2\left(\bar{d} + \overline{\beta_0}\bar{d}\right) - \mathrm{Cl}_2\left(\bar{d} - \overline{\beta_0}\bar{d}\right) \\ &\quad = \bar{d}\, ln\left|csc\frac{\bar{d} + \overline{\beta_0}\bar{d}}{2} sin\frac{\bar{d} - \overline{\beta_0}\bar{d}}{2}\right| + \frac{\bar{d}^2\left[cos\left(\overline{\beta_0}\bar{d}\right) - cos\,\bar{d}\right]}{csc\left(\overline{\beta_0}\bar{d}\right)},\end{aligned} \tag{S2}$$

which is an implicit function for the stationary point $\beta_0 = \bar{\beta}_0 k$ of both frequency $\omega = ck$ and the lattice constant $d = \bar{d}/k$. We clearly see an asymptote around $\bar{d} \approx 1.517$ (red dash-dot line)

in the $\bar{\beta}_0$-$\bar{d}$ plot as shown in Fig. S1, which implies that the stationary point inside the first Brillouin zone exists only for sufficiently compact arrays where $kd < 1.517$. For the analytical quantification of this condition, the reader is referred to Ref. [1].

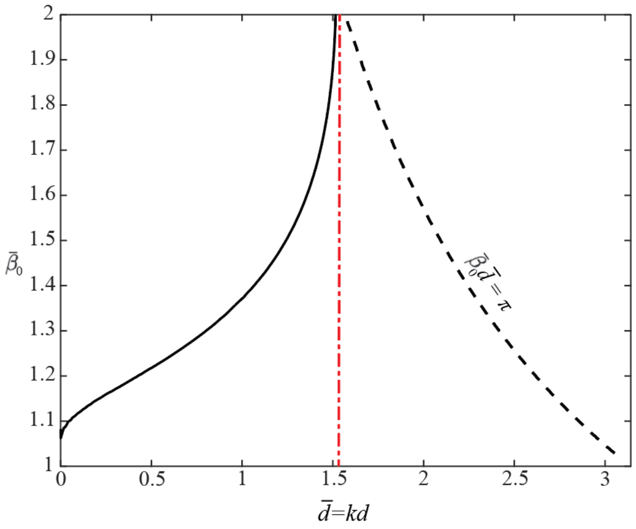

FIG. S1. The locus of the stationary point (zero group velocity) as a function of lattice compactness. The solid black line shows the dependence of $\overline{\beta_0}$ on $\bar{d}$, whose asymptote is drawn by the red dash-dot line ($\bar{d} = kd \approx 1.517$). The black dashed line depicts the edge of the first Brillouin zone, which is a trivial solution.

2. Green's functions and their singularities

The Green's functions involved in Eq. (4) in the main text can be derived from the local fields at any two adjacent dipoles' locations. Without loss of generality, we choose $\mathbf{p}_1$ at the origin in Fig. 1 in the main text as our interested one. Then, the local field at the origin is

$$\boldsymbol{E}_{loc,1} = \boldsymbol{E}_{11} + \boldsymbol{E}_{12}, \tag{S3}$$

where $\mathbf{E}_{11}$ and $\mathbf{E}_{21}$ are the contributions from the sequent dipole moments of $\mathbf{p}_1$ [except $\mathbf{p}_1$ itself, as in Fig. S2(a)]and those of $\mathbf{p}_2$ [as in Fig. S2(b)] with Bloch phase accumulation, respectively.

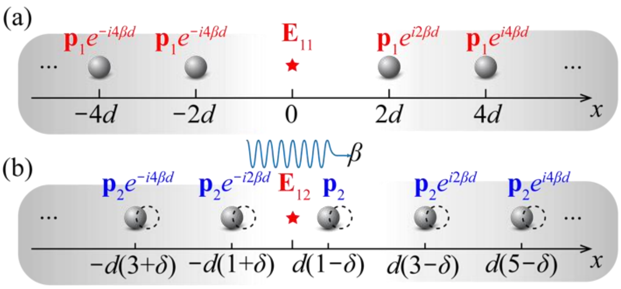

FIG. S2. The contributions of local fields at the origin (denoted by the red star). $\mathbf{E}_{12}$ in (a) represents the contribution from the sequent dipole moments of $\mathbf{p}_1$ except itself, while $\mathbf{E}_{21}$ in (b) represents that from $\mathbf{p}_2$ and its sequence.

For the transverse polarization, the transverse component of the local field contributed by the sequence of $\mathbf{p}_1$ is simply the summation of the contributions of every involved dipole. It reads [2]

$$\begin{aligned} E_{11} &= -\frac{1}{4\pi\varepsilon_0}\sum_{n\neq 0}\frac{1-i2|n|kd-(2nkd)^2}{(2|n|d)^3}p_1 e^{i2n\beta d}e^{i2|n|kd} \\ &= -\frac{p_1 k^3}{4\pi\varepsilon_0}\left[\frac{f_3(\beta/k,kd)}{(2kd)^3}-\frac{if_2(\beta/k,kd)}{(2kd)^2}-\frac{f_1(\beta/k,kd)}{2kd}\right] \end{aligned} \tag{S4}$$

with $f_\ell(\beta/k,kd) = \mathrm{Li}_\ell\left[e^{i2(1+\beta/k)kd}\right] + \mathrm{Li}_\ell\left[e^{i2(1-\beta/k)kd}\right]$. $\mathrm{Li}_\ell(\cdot)$ is the polylogarithm of ℓ-th order [3]. Thus, we obtain $G_{11} = E_{11}/p_1$, which reads,

$$G_{11} = -\frac{k^3}{4\pi\varepsilon_0}\left[\frac{f_3(\beta/k,kd)}{(2kd)^3}-\frac{if_2(\beta/k,kd)}{(2kd)^2}-\frac{f_1(\beta/k,kd)}{2kd}\right]. \tag{S5}$$

Since the two particles are identical, $G_{11} = G_{22}$ evidently. The off-diagonal term G_{12} (or G_{21}) can be derived similarly by summing over all the field contributions from corresponding dipoles shown in Fig. S2(b). In detail, $G_{21}(\beta) = G_{12}(-\beta)$ as required by the reciprocity, and $G_{12} = E_{12}/p_2$, which reads,

$$G_{12} = -\frac{1}{4\pi\varepsilon_0}\left[\sum_{n=0}^{+\infty}\frac{1-i(2n+x_{21})kd-(2n+x_{21})^2(kd)^2}{(2n+x_{12})^3d^3}e^{-i(2n+2)\beta d}e^{i(2n+x_{21})kd} + \sum_{n=0}^{+\infty}\frac{1-i(2n+x_{12})kd-(2n+x_{12})^2(kd)^2}{(2n+x_{12})^3d^3}e^{i2n\beta d}e^{i(2n+x_{12})kd}\right] = -\frac{k^3}{4\pi\varepsilon_0}\left[\frac{h_3(\beta/k,kd,\delta)}{(2kd)^3}-\frac{ih_2(\beta/k,kd,\delta)}{(2kd)^2}-\frac{h_1(\beta/k,kd,\delta)}{2kd}\right], \tag{S6}$$

with

$$h_\ell(\beta/k,kd,\delta) = e^{ix_{12}kd}\Phi\left[e^{i2(1+\beta/k)kd},\ell,x_{12}/2\right] + e^{-ix_{12}kd}e^{i2(1-\beta/k)kd}\Phi\left[e^{i2(1-\beta/k)kd},\ell,x_{21}/2\right]. \tag{S7}$$

$x_{21} = 1-\delta$ and $x_{12} = 1+\delta$ represent the relative distance between two adjacent dipoles inside the unit cell (e.g., $\mathbf{p}_1$ and $\mathbf{p}_2$) and intra-cell (e.g., $\mathbf{p}_2$ and $\mathbf{p}_1 e^{i2\beta d}$), respectively. $\Phi(z,\ell,x) = \sum_{n=0}^{\infty} z^n(n+x)^{-\ell}$ is the Lerch transcendental. Similar to the Hurwitz zeta function, the Lerch transcendental have branch-point singularities at $x = 0,-1,-2,\ldots$ [4]-[5]. Thus, we require $\delta \neq \pm 1$. This requirement is automatically satisfied because the distance between two classical particles cannot be smaller than the sum of their radii, which leads to $|\delta| \leq |1-d/(2a)|$. Besides, the derived Green's functions can be analytically continued to the whole complex plane of ω. The Green's functions for longitudinal polarization can be derived similarly, which read,

$$G_{11} = \frac{k^3}{2\pi\varepsilon_0}\left[\frac{f_3(\beta/k,kd)}{(2kd)^3}-\frac{if_2(\beta/k,kd)}{(2kd)^2}\right], \tag{S8}$$

$$G_{12} = \frac{k^3}{2\pi\varepsilon_0}\left[\frac{h_3(\beta/k,kd,\delta)}{(2kd)^3}-\frac{ih_2(\beta/k,kd,\delta)}{(2kd)^2}\right]. \tag{S9}$$

The normalized scalar Green's functions presented in the main text are then $\bar{G}_{i,j} = 6\pi\varepsilon_0 G_{i,i}/k^3$. To write Eqs. (S5)-(S9) compactly, we leverage the scalar product of dipole polarization vector and that of the array's orientation as presented in Eq. (1) in the main text.

Notice that the light line $\beta = k$ is a branch point of both polylogarithms, Lech transcendental functions [5], and further of all Greens functions [Eqs. (S5)-(S9)], which gives rise to the branches of non-modal solutions on the light line as discussed in the main text. Meanwhile, the light line is also an essential singularity of Eqs. (S5) and (S6), because they involve the first-order polylogarithm $\mathrm{Li}_1(z)$ and Lerch transcendental $\Phi(z, 1, x)$. When $z = 1$, i.e., $\beta = k$, both of them behave as an ill-defined harmonic series and lead to an essential singularity. It implies thar in the scenarios of transverse polarization, $\beta/k = 1$ is not only a branch point but also a diverging essential singularity of the Green's functions, which explains the discontinuities in $\mathrm{Im}(\omega)$in Fig. 2(e) and Fig. 4(e) in the main text.

3. Trivial topological knot in non-Hermitian plasmonic arrays

To investigate the topological property of the knot emerging from self-folding a highly nonlocal branch, we consider a plasmonic array with dielectric loss. For example, we choose the collision rate to be $\gamma = 10^{-6}\omega_0$ in the Drude model. In this case, the electric polarizability satisfies

$$Im\{\alpha_{ee}^{-1}\} = -\frac{k^3}{6\pi\varepsilon_0 a^3} - \frac{3}{4\pi a^3}\frac{Im\{\varepsilon\}}{Im(\varepsilon)^2 + (Re\{\varepsilon\} - \varepsilon_0)^2}. \qquad \text{(S10)}$$

where $\varepsilon = \varepsilon_0[1 - 3\omega_0^2/(\omega^2 + i\gamma\omega)]$. As in the main text, we set β to be a real quantity while solving complex solutions of ω. Using the same geometrical parameters as in Fig. 2, we calculate the band structure for this lossy plasmonic array in the transverse polarization, as

shown in Fig. S3. The braid word for the non-Hermitian Bloch band in Fig. S3(a) is $\tau_1\tau_1^{-1}$, implying a trivial topological knot, known as unlink [6]. Alternatively, we can also plot the band structure in the polar coordinate with the eigenfrequency $\mathrm{Re}(\omega)$ in the radial direction and the Bloch phase $2\beta d$ being the azimuthal angles. As shown in Fig. S3(b), the polar plot also demonstrates an unlink with two disconnected circles.

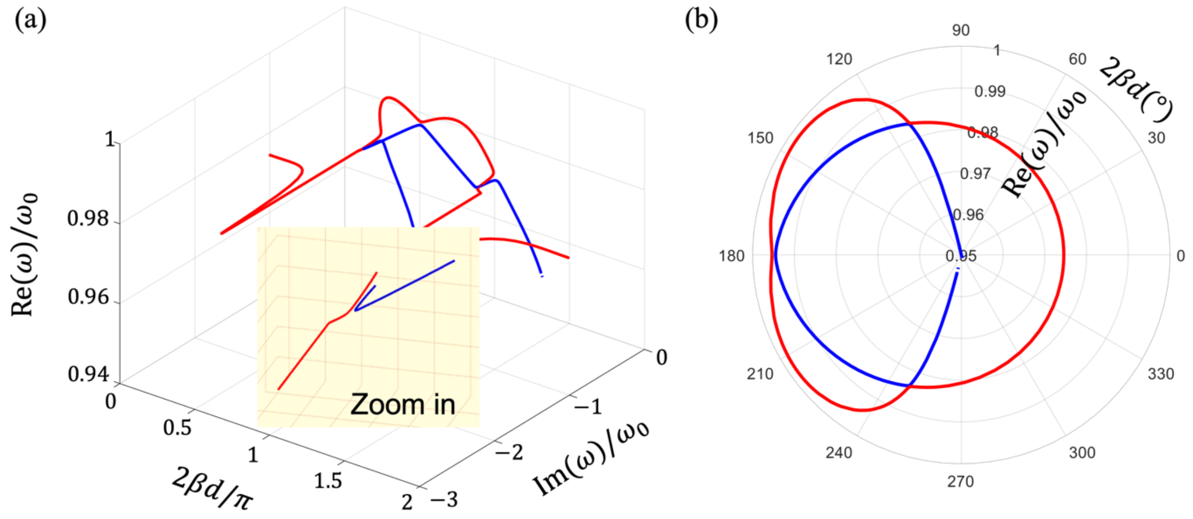

FIG. S3. Bloch band structure for the transversely polarized lossy plasmonic array with Drude collision rate $\gamma = 10^{-6}\omega_0$ in the (a) β-complex ω space and in the (b) polar coordinate.

4. Band knots in the presence of material loss

The typical collision rates of realistic plasmonic materials are in the order of $\gamma{\sim}10^{-3}\omega_0$ [7]. Here we numerically study how such level of material loss affects the dispersion braiding and associated band knots described in the main text. Considering a more practical scenario to demonstrate the robustness of the band knots, we choose the same geometry accounted for the green dispersion curve shown in Fig. 4(a) in the main text, where the relative translation perturbation δ is as large as 0.1. Figure S4(a) shows the dispersion diagram of such a perturbed array, yet under material loss described by a Drude collision rate $\gamma = 10^{-3}\omega_0$. The error bars denote twice the imaginary part of the eigenfrequency, indicating the linewidth of practically

measurable optical spectra, e.g., reflectance, transmittance or absorption. As long as the linewidths of the two modes (red and blue curves) do not overlap, we may safely assume that these two modes are distinguishable in the spectrum. As shown in Fig. S4(a), the braiding and associated band knots formed by the two eigenmodes are clearly observable in the presence of this level of material loss. If we increase γ to $5 \times 10^{-3}\omega_0$, as shown in Fig. S4(b), the spectra of both modes broaden.

To further verify the realizability of the proposed band knots using realistic materials, we take silver as an example to construct the perturbed arrays, whose permittivity can be approximated by $\varepsilon/\varepsilon_0 = 5 - \omega_p^2/(\omega^2 + i\omega\gamma)$ with $\omega_p = 2\pi \times 2175$ THz and $\gamma = 2\pi \times 4.35$ THz [7]. The radius of the spheres is set to $a = 20$ nm and the unperturbed periodicity $d = 60$ nm. Under the perturbation extent $\delta = 0.1$, i.e., translating every other two spheres by 6 nm, the dispersion diagrams of the perturbed array are shown in Fig. S4(c). The anti-crossing band knots now become less visible, due to significant broadening of the spectra. However, we can recover the braiding and band knots phenomena by engineering the perturbations. For instance, we may simply loosen the braid to some extent by increasing the relative translation distance, as discussed around Fig. 4(a) in the main text, such that the spectrum separation of the two modes is wide enough to observe the anti-crossing again. As shown in Fig. S4(d), the band knot becomes more obvious again once we increase the perturbation to $\delta = 0.15$.

In conclusion, the dispersion braiding and associated band knots are not hindered by material loss, and hence also by random disorders from fabrication imperfections [8]. In fact,

we can engineer the perturbations discussed in the main text to achieve the desired braiding in reciprocal space, according to different levels of material loss and imperfections.

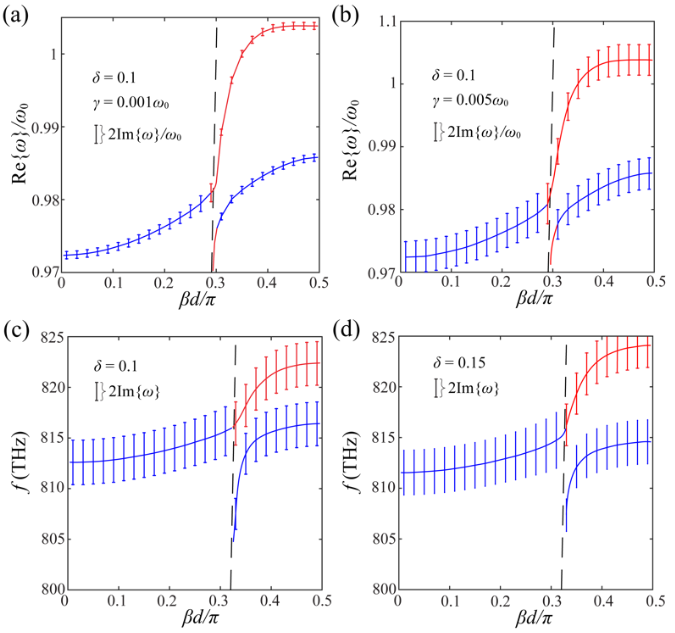

FIG. S4. (a,b) Dispersion diagrams of the perturbed arrays of transversely polarized plasmonic spheres, under different level of material loss denoted by the collision rate γ. (c-d) Those for the perturbed arrays, under different relative translation distance δ, comprising realistic silver spheres of radius 20 nm and unperturbed periodicity 60 nm.

References

[1] A. Alù and N. Engheta, "Theory of linear chains of metamaterial/plasmonic particles as subdiffraction optical nanotransmission lines," *Physical Review B*, vol. 74, no. 20, pp. 1–18, 2006, doi: 10.1103/PhysRevB.74.205436.

[2] J. D. Jackson, *Classical Electrodynamics*. Hoboken: John Wiley & Sons, 1999.

[3] L. Lewin, *Polylogarithms and Associated Functions*. New York: Elsevier North-Holland. Inc., 1981.

[4] H. Bateman, *Higher transcendental functions*, vol. 1. McGraw-Hill Book Company, 1953.

[5] L. Vepštas, "An efficient algorithm for accelerating the convergence of oscillatory series, useful for computing the polylogarithm and Hurwitz zeta functions," *Numerical Algorithms*, vol. 47, no. 3, pp. 211–252, 2008, doi: 10.1007/s11075-007-9153-8.

[6] H. Hu and E. Zhao, "Knots and Non-Hermitian Bloch Bands," *Physical Review Letters*, vol. 126, no. 1, p. 010401, 2021, doi: 10.1103/PhysRevLett.126.010401.

[7] P. B. Johnson and R. W. Christy, "Optical Constants of the Noble Metals," *Phys. Rev. B*, vol. 6, no. 12, pp. 4370–4379, Dec. 1972, doi: 10.1103/PhysRevB.6.4370.

[8] A. Alù and N. Engheta, "Effect of small random disorders and imperfections on the performance of arrays of plasmonic nanoparticles," *New J. Phys.*, vol. 12, no. 1, p. 013015, Jan. 2010, doi: 10.1088/1367-2630/12/1/013015.
